# Hypochlorous acid solution is a potent antiviral agent against SARS‐CoV‐2

**DOI:** 10.1111/jam.15284

**Published:** 2021-09-17

**Authors:** Noritoshi Hatanaka, Mayo Yasugi, Tomoko Sato, Masafumi Mukamoto, Shinji Yamasaki

**Affiliations:** ^1^ Graduate School of Life and Environmental Sciences Osaka Prefecture University Osaka Japan; ^2^ Asian Health Science Research Institute Osaka Prefecture University Osaka Japan; ^3^ Osaka International Research Center for Infectious Diseases Osaka Prefecture University Osaka Japan; ^4^ Research Laboratory Local Power Co., Ltd. Akita Japan

**Keywords:** antiviral activity, COVID‐19, hypochlorous acid, SARS‐CoV‐2, TGEV

## Abstract

**Aim:**

A novel coronavirus, termed severe acute respiratory syndrome coronavirus 2 (SARS‐CoV‐2) suddenly appeared in Wuhan, China, and has caused pandemic. In this study, we evaluated antiviral activity of purified hypochlorous acid (HClO) against coronaviruses such as SARS‐CoV‐2 and transmissible gastroenteritis virus (TGEV) responsible for pig diseases.

**Materials and Results:**

In a suspension test, 28.1 ppm HClO solution inactivated SARS‐CoV‐2 in phosphate‐buffered saline with the reduction of 10^4^ of 50% tissue culture infectious dose per ml (TCID_50_ per ml) within 10 s. When its concentration increased to 59.4 ppm, the virus titre decreased to below the detection limit (reduction of 5 logs TCID_50_) within 10 s even in the presence of 0.1% foetal bovine serum. In a carrier test, incubation with 125 ppm HClO solution for 10 min or 250 ppm for 5 min inactivated SARS‐CoV‐2 by more than 4 logs TCID_50_ per ml or below the detection limit. Because the titre of TGEV was 10‐fold higher, TGEV was used for SARS‐CoV‐2 in a suspension test. As expected, 56.3 ppm HClO solution inactivated TGEV by 6 logs TCID_50_ within 30 s.

**Conclusions:**

In a carrier test, 125 ppm HClO solution for 10 min incubation is adequate to inactivate 4 logs TCID_50_ per ml of SARS‐CoV‐2 or more while in a suspension test 56.3 ppm HClO is adequate to inactivate 5 logs TCID_50_ per ml of SARS‐CoV‐2 when incubated for only 10 s regardless of presence or absence of organic matter.

**Significance and Impact of the Study:**

Effectiveness of HClO solution against SARS‐CoV‐2 was demonstrated by both suspension and carrier tests. HClO solution inactivated SARS‐CoV‐2 by 5 logs TCID_50_ within 10 s. HClO solution has several advantages such as none toxicity, none irritation to skin and none flammable. Thus, HClO solution can be used as a disinfectant for SARS‐CoV‐2.

## INTRODUCTION

Coronaviruses (CoVs) are enveloped, single‐stranded RNA viruses. Some of these viruses are known to cause respiratory, gastrointestinal and neurologic diseases in human, such as HCoVs‐229E, HCoVs‐OC43, HCoVs‐NL63, HCoVs‐HKU1, severe acute respiratory syndrome CoV (SARS‐CoV) and Middle East respiratory syndrome CoV (MERS‐CoV) (Al‐Omari et al., 2019; Doremalen et al., 2020). Except SARS‐CoV and MERS‐CoV, remaining four CoVs cause common cough and cold symptoms in human. On the other hand, SARS‐CoV caused an outbreak of severe respiratory syndrome in 2002–2003 in China and MERS‐CoV caused similar disease in the Middle East in 2012 (Wu et al., 2020).

At the end of 2019, a novel CoV has been isolated from patients with pneumonia of unknown cause in Wuhan, China and was named severe acute respiratory syndrome CoV‐2 (SARS‐CoV‐2) (Al‐Omari et al., 2019; Zhu et al., 2020). SARS‐CoV‐2 has been responsible for COVID‐19 pandemic and has spread to six continents soon after the discovery of the virus. As of August 27, 2021, more than 214 million confirmed cases with 4.47 million deaths were reported to WHO (World Health Organization, 2021). Symptoms of COVID‐19 are varying from asymptomatic to severe pneumonia and death. Even though the mortality rate of COVID‐19 is lower than that of SARS and MERS, a number of countries including Japan, declared emergency and stopped social activities.

It is considered that the major infection routes of SARS‐CoV‐2 are respiratory droplet transmission and through contact (Jin et al., 2020). Therefore, it is important to inactivate the virus in droplet and on surfaces to prevent the transmission of the virus to human. Hypochlorous acid (HClO), the major reactive oxygen species produced by macrophages and neutrophils, played a role as a disinfectant to kill invading microorganisms (Miller & Britigan, 1997). It has been shown that HClO affects oxidative protein folding and in bacteria it leads to aggregation of vital proteins such as redox‐regulated chaperone Hsp33, which might result in antimicrobial activity (Winter et al., 2008). Recently, it has been reported that a sprayable acid‐oxidizing solution containing HClO inactivated SARS‐CoV‐2 by more than 99.8% within 1 min (Giarratana et al., 2021). However, reduction rate of the virus titre was only 99.8% or more. Therefore, in this study, we examined the antiviral activity of highly purified HClO solution against SARS‐CoV‐2 and how much concentration and time are needed the HClO solution to inactivate 10^4^ or more numbers of SARS‐CoV‐2.

## MATERIALS AND METHODS

### Cell culture

VeroE6/TMPRSS2 (Matsuyama et al., 2020) and Vero cells were used for cultivation of SARS‐CoV‐2 and transmissible gastroenteritis virus (TGEV), respectively. Both cell lines were incubated at 37℃ in a 5% CO_2_ humidified incubator (ESPEC CORP.). VeroE6/TMPRSS2 cell line was purchased from Japanese Collection of Research Bioresources. The cells were cultured in Dulbecco's modified eagle medium, low glucose, pyruvate (DMEM; Thermo Fisher Scientific Inc.) supplemented with 5% heat inactivated foetal bovine serum (FBS; Thermo Fisher Scientific Inc.) and 1 mg/ml of G418 (Nacalai Tesque Inc.). Vero cells were cultured in minimum essential medium (MEM; Nissui Pharmaceutical Co. Ltd) supplemented with 5% FBS, 1% GlutaMax and 1% antibiotic‐antimycotic (Thermo Fisher Scientific Inc.).

### Virus preparation

One‐hundred‐and‐forty thousand cells of VeroE6/TMPRSS2 or Vero cells were cultured in a 25 cm^2^‐cell culture flask for 16 h, in a 5% CO_2_ humidified incubator. The cells were infected with a multiplicity of infection of 0.001 of SARS‐CoV‐2 JPN/TY/WK‐521 or TGEV Vero cell adapted TO‐163 strain (Ishii et al., 1992) in DMEM supplemented with 2% FBS and 1 mg/ml of G418 or MEM supplemented with 1% FBS, respectively, and incubated for 48–96 h. After cytopathic effects (CPEs) was observed, the spent culture medium was harvested and centrifuged at 1,600 *g* for 5 min, and the supernatant fraction containing virus particles was collected and further concentrated as described below.

One gram of polyethylene glycol (PEG) 6000 and 233 mg of NaCl (Nacalai Tesque Inc.) were added to 10 ml of collected viral solution, and the solution was incubated at 4℃ for 16 h. After that, the solution was centrifuged at 20,000 *g* at 4℃ for 10 min, and the pellet was collected and suspended in 1 ml of phosphate‐buffered saline (PBS). Experiments with live SARS‐CoV‐2 virus were conducted at the bio‐safety level 3 laboratory in Osaka Prefecture University after obtaining the permission from the bio‐risk committee of Osaka Prefecture University.

### Titration of virus

About 2.5 × 10^4^ cells per 100 µl of VeroE6/TMPRSS2 or Vero cells were seeded in a 96‐well plate and cultured for 16 h in their respective medium. Culture medium was removed and 100 µl of 10‐fold serially diluted viral solution in DMEM supplemented with 2% FBS and 1 mg/ml of G418 for SARS‐CoV‐2 or MEM supplemented with 1% FBS for TGEV was added. The infected VeroE6/TMPRSS2 and Vero cells were cultured for 72 and 96 h, respectively. To calculate infective dose, four wells were used for each dilution including control, and experiments were carried out three times, independently. Cells were fixed with methanol (Nacalai Tesque Inc.) and stained with 0.5% crystal violet stain. Then, 50% tissue culture infective dose (TCID_50_) per millilitre was calculated. The detection limit was ≤1.5 logs TCID_50_ per ml.

### Antiviral activity of HClO solution

HClO solution (iPOSH: Local Power) used in this study is a mixture of HClO and hypochlorite ion. HClO in iPOSH was purified by ion exchange method using sodium hypochlorite (NaClO) solution, by which residual sodium ions were removed. Since some of HClO can be dissociated to hypochlorite ion in water, iPOSH contains not only HClO but also hypochlorite ion. Sixty microliters of concentrated SARS‐CoV‐2 in PBS or 30 µl of the virus cultured in DMEM supplemented with 2% FBS, whose titre was around 6.6 logs TCID_50_ per ml, were mixed with 540 or 570 µl of different concentrations (250, 125, 62.5, 31.3 ppm) of HClO solution in 1.5 ml polypropylene tube. Then, the mixture was incubated at room temperature (25°C) for 10 or 30 s and 1, 3 or 5 min. After incubation, 30 µl of 0.1 M sodium thiosulfate, 60 µl of 10 times concentrated medium (10× DMEM [Nissui Pharmaceutical Co. Ltd], 20% FBS and 10 mg/ml G418 disulfate aqueous solution for SARS‐CoV‐2 or 10× MEM [Nissui Pharmaceutical Co. Ltd], 10% FBS, 6 µl of GlutaMax and 6 µl of anti‐anti [Thermo Fisher Scientific Inc.] for TGEV) were immediately added, and titration was done as described above.

### Carrier test

A carrier test was performed as described previously (Sattar et al., 2003). Briefly, 10 µl each of virus solution was spotted in eight places on a slide glass and dried for 50 min at room temperature (25°C). After drying, 50 µl of several concentrations (250, 125 or 62.5 ppm) of HClO solution were dropped on the slide glass and kept for 30, 60 s, 5 or 10 min. Then, HClO was neutralized with 50 µl of medium (2× DMEM, 4% FBS, 2 mg/ml of G418 and 200 mmol m^−3^ sodium thiosulfate) for each drop. After neutralization, the SARS‐CoV‐2 virus was collected by a cell scraper (NIPPON Genetics Co., Ltd, Tokyo, Japan), and virus titre was determined as described before.

### Statistical analysis

Statistical analyses were performed using Microsoft Excel 2016 (Microsoft). Error bars show standard deviations. *p*‐values were calculated with Student's *t*‐test using paired, two‐tailed distribution. It considered statistically significant when *p*‐value showed less than 0.05 in difference of data.

## RESULTS

### Antiviral activity of HClO solution against SARS‐CoV‐2

**FIGURE 1:**
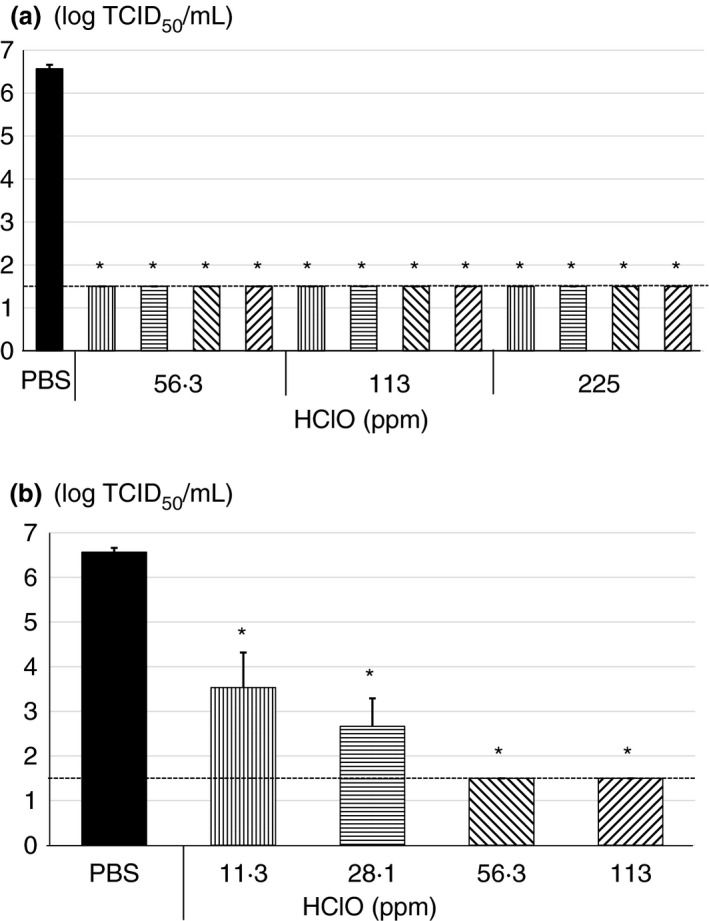
Antiviral activity of hypochlorous acid (HClO) against severe acute respiratory syndrome coronavirus 2 (SARS‐CoV‐2). Concentrated SARS‐CoV‐2 by polyethylene glycol was treated with (a) 56.3, 113 or 225 ppm of HClO for 0.5 (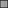), 1 (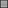), 3 (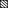) or 5 (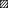) min, and (b) 11.3 (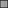), 28.1 (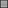), 56.3 (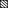) or 113 (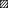) ppm of HClO for 10 s followed by determination of virus titer with 50% tissue culture infective dose. All data represent the mean + SD from three independent experiments. Dot line indicates detection limit for experiment. * indicates that values are significantly different between phosphate‐buffered saline (PBS) and each experimental condition

To examine the antiviral activity of HClO solution, concentrated SARS‐CoV‐2 (approximately 7.0 logs TCID_50_ per ml) was incubated with various concentrations (56.3, 113 or 225 ppm) of HClO solution for 30 s to 5 min (Figure 1a). When SARS‐CoV‐2 was incubated with 10 mmol m^−3^ PBS at pH 7.4 for 5 min, the virus titre was 6.6 ± 0.1 logs TCID_50_ per ml. However, when incubated with 56.3, 113 or 225 ppm HClO solution for 30 s to 5 min, the virus titre was reduced to be below the detection limit (≤1.5 logs TCID_50_ per ml), indicating that treatment with 56.3 ppm HClO solution for 30 s is adequate to reduce the titre by 5 logs TCID_50_ per ml (Figure 1a).

To determine the limit of antiviral activity of the HClO solution, the virus was incubated with lower concentrations of HClO solution for only 10 s. When the virus was incubated with 56.3 or 113 ppm HClO solution for 10 s, the virus titre decreased to below the detection limit (≤1.5 log TCID_50_ per ml). However, when the virus was incubated with 11.3 or 28.1 ppm HClO solution for 10 s, the virus titre was decreased from 6.6 ± 0.1 to 3.5 ± 0.8 or 2.7 ± 0.6 logs TCID_50_ per ml, indicating that HClO solution reduced 3–4 logs TCID_50_ or more SARS‐CoV‐2 within 10 s in a dose‐dependent manner and 56.3 and 113 ppm inactivated more than 5 logs TCID_50_ of SARS‐CoV‐2 (Figure 1b).

### Antiviral activity of HClO solution against TGEV

**FIGURE 2:**
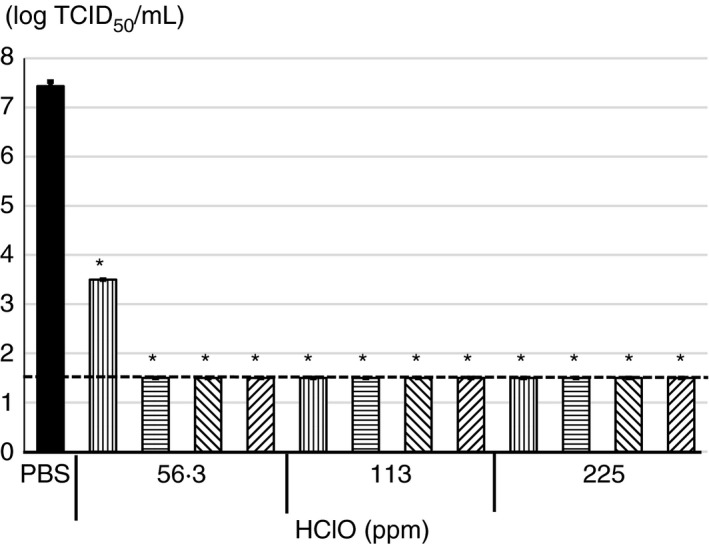
Antiviral activity of various concentrations of hypochlorous acid (HClO) against transmissible gastroenteritis virus (TGEV). Concentrated TGEV by polyethylene glycol was treated with 56.3, 113 or 225 ppm of HClO for 0.5 (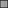), 1 (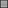), 3 (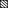) or 5 (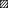) min followed by determination of virus titer with 50% tissue culture infective dose. All data represent the means + SD from three independent experiments. Dot line indicates detection limit for experiment. * indicates that values are significantly different between phosphate‐buffered saline (PBS) and each experimental condition

Since virus titre of SARS‐CoV‐2 did not reach to 7.0 logs TCID_50_ per ml and 56.3 or 113 ppm HClO solution could effectively reduce the virus titre to below detection limit, we, therefore, further examined the antiviral activity of the HClO solution with concentrated TGEV, which is also one of the CoVs and can reach to more than 7.0 logs TCID_50_ per ml. When TGEV was incubated with PBS for 5 min, the virus titre was 7.4 ± 0.1 logs TCID_50_ per ml. However, the virus titre reduced by approximately 6 logs TCID_50_ and to the detection limit (≤1.5 logs TCID_50_ per ml) when incubated with 56.3, 113 or 225 ppm HClO solution for 30 s to 5 min, except with 56.3 ppm for 30 s (Figure 2).

### Antiviral activity of HClO solution against SARS‐CoV‐2 in the presence of 0.1% FBS

**FIGURE 3:**
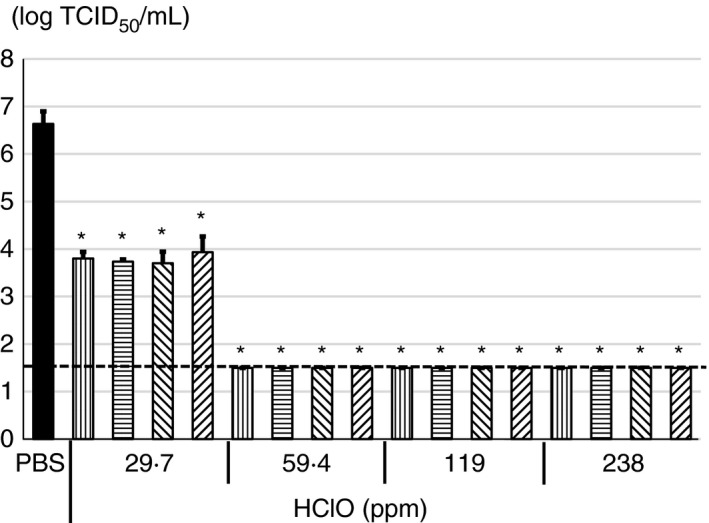
Antiviral activity of various concentrations of hypochlorous acid (HClO) against severe acute respiratory syndrome coronavirus 2 (SARS‐CoV‐2) in the presence of 0.1% FBS. SARS‐CoV‐2 cultured in DMEM supplemented with 2% FBS was treated with 29.7, 59.4, 119 or 238 ppm of HClO for 10 (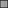), 30 (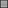) s, 1 (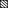) or 3 (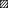) min followed by determination of virus titer with 50% tissue culture infective dose. All data represent the means + SD from three independent experiments. Dot line indicates detection limit for experiment. * indicates that values are significantly different between phosphate‐buffered saline (PBS) and each experimental condition

Since saliva is the most important infection source of SARS‐CoV‐2, antiviral activity of the HClO solution was evaluated under organic matter rich condition. It has been reported that protein concentration of saliva is 1.12 ± 0.39 or 1.11 ± 0.42 mg/ml, which was measured by Lowry method or BCA protein assay, respectively (Jenzano et al., 1986). Therefore, the virus was incubated with various concentrations of HClO solution in MEM supplemented with 0.1% FBS as a final concentration. Because, 0.1% FBS contain almost same concentration of proteins as found in saliva i.e., 1.1 mg/ml as determined by the Bradford protein assay (data not shown). When the virus was incubated with 59.4, 119 or 238 ppm HClO solution for 10 s to 3 min in the presence of 0.1% FBS, the virus titre was decreased from 6.6 ± 0.1 logs TCID_50_ per ml to below the detection limit (≤1.5 logs TCID_50_ per ml). When the virus was incubated with 29.7 ppm HClO solution under the same condition, the virus titre was decreased to 3.7–3.9 logs TCID_50_ per ml (Figure 3).

### Antiviral activity of HClO solution against SARS‐CoV‐2 in a carrier test

**FIGURE 4:**
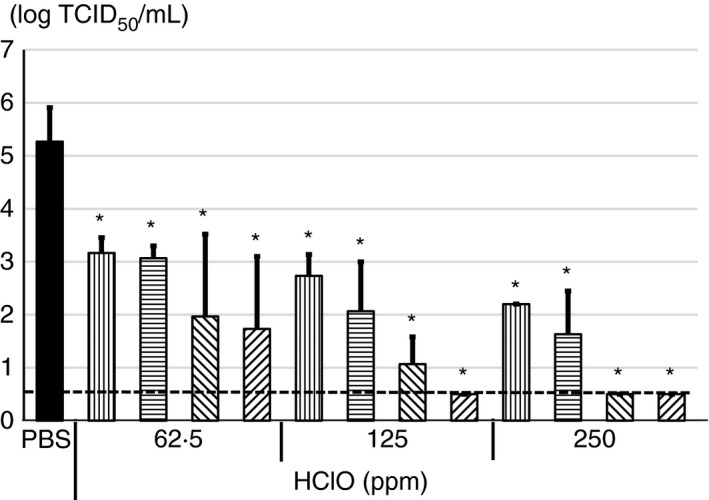
Antiviral activity of various concentrations of hypochlorous acid (HClO) against severe acute respiratory syndrome coronavirus 2 (SARS‐CoV‐2) in carrier test. 10 µl of concentrated SARS‐CoV‐2 was spotted on slide glass and dried. Then, 50 µl of 62.5, 125 or 250 ppm HClO was dropped on the slide glass and kept for 30 (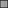), 60 (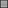) s, 5 (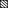) or 10 (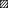) min followed by determination of virus titer with 50% tissue culture infective dose. All data represent the means + SD from three independent experiments. Dot line indicates detection limit for experiment. * indicates that values are significantly different between phosphate‐buffered saline (PBS) and each experimental condition

To examine further utility of iPOSH in real situation, antiviral activity of HClO solution was evaluated by a carrier test. The SARS‐CoV‐2 virus showing 5.3 ± 0.6 logs TCID_50_ per ml after adding and drying on slide glass for 50 min were used in a carrier test. When SARS‐CoV‐2 on slide glass was treated with 62.5 ppm HClO solution for 5 or 10 min, the virus titre was decreased by approximately 3 logs TCID_50_. On the other hand, when SARS‐CoV‐2 was treated with 125 or 250 ppm HClO solution for 5 or 10 min, the virus titre was decreased by approximately 4 logs TCID_50_. Especially, when SARS‐CoV‐2 was treated with 125 ppm for 10 min or 250 ppm for 5 or 10 min, the virus titre was decreased to below the detection limit (≤0.5 log TCID_50_ per ml) (Figure 4).

## DISCUSSION

It has been reported that infectious SARS‐CoV‐2 could be detected from surfaces of plastic and stainless steel after 72 h of its application (Doremalen et al., 2020). Even though, it is at present unclear that how many SARS‐CoV‐2 viruses as infective dose are required for causing the disease in human, inactivation of SARS‐CoV‐2 is most important to prevent transmission of the virus to human. WHO has recommended 1,000 ppm NaClO or 70% ethanol for disinfection of the surfaces (World Health Organization, regional office for the Western Pacific, 2020). Furthermore, the National Institute of Technology and Evaluation (NITE), Japan, has reported that 35 ppm or more HClO could be also used as a disinfectant against SARS‐CoV‐2. However, if HClO is generated by the electrolysis method, available chlorine concentration is less than 60 ppm and HClO in the solution is unstable. Because HClO generated by electrolysis contains extra NaCl and other cationic ions, which are responsible for degradation of HClO in the water (data not shown). Therefore, in this study, we evaluated purified HClO solution, (iPOSH: Local Power), which was prepared by an ion exchange method using NaClO solution, in suspension and carrier tests. HClO solution prepared by the ion exchange method can reach to a high‐concentration solution up to 1,000 ppm and may be kept at room temperature (20°C) for a long time such as a year under dark condition because of its high purity (data not shown). Eighty and six percent of effective chlorine concentration (250 ppm became 215 ppm) remained after 6 months and 80% remained after a year. The advantage of HClO solution is that it can be applicable for not only disinfecting substances but also as an effective hand sanitizer. Because it has been shown that there is no acute oral toxicity in mice by using 500 ppm purified HClO solution (Japan Food Research Laboratory). Similarly, iPOSH containing 250 ppm HClO did not cause skin irritation in humans by open patch test (Souken Co., Ltd.). Furthermore, the US Food and Drug Administration has approved the use of HClO solution for eye and dental spray application (Nguyen et al., 2021). Thus, HClO solution has several advantages in comparison with alcohol‐based disinfectant and NaClO: it is none toxic, none irritation to skin and none flammable.

In a suspension test, when SARS‐CoV‐2 (6.6 ± 0.1 log TCID_50_ per ml) was incubated with 59.4 ppm HClO solution for only 10 s or more, CPE was not observed regardless of presence or absence of organic matter in the viral solution (Figures 1 and 3). Although TGEV showed higher titre (7.4 ± 0.1 logs TCID_50_ per ml) than SARS‐CoV‐2, CPE was not observed when TGEV was incubated with 56.3 ppm HClO for 1 min or more (Figure 2). However, when SARS‐CoV‐2 was incubated with 29.7 ppm HClO solution for 3 min under organic matter–rich condition, CPE was observed (Figure 3). It should be noted that effective chlorine concentration was maintained after incubation time when PEG was not present. However, the effective chlorine concentration after incubation time was not able to measure in the presence of PEG (data not shown). Most probably effective chlorine concentration could be maintained even in the presence of PEG after incubation time. Nevertheless, these results indicate that in a suspension test, at least 1 min incubation with 59.4 ppm or more HClO solution is necessary to inactivate 5 logs or more TCID_50_ per ml of SARS‐CoV‐2 or TGEV.

On the contrary, in a carrier test, 5 min incubation with 125 ppm HClO was needed to decrease virus titre by more than 4 logs TCID_50_ per ml (Figure 4). Moreover, 10 min incubation with 125 ppm HClO and 5 min incubation with 250 ppm HClO could decrease virus titre below the detection limit (Figure 4). Wölfel et al. (2020) reported that the average copy number of SARS‐CoV‐2 RNA in sputum samples from patients with COVID‐19 is 7.0 × 10^6^ copies per ml. Therefore, SARS‐CoV‐2 in droplets from patients might be effectively inactivated if 125 ppm or more HClO solution is applied.

Hakim et al. (2015) have reported that spraying of 50 ppm HClO solution to a low pathogenic avian influenza virus (AIV), which is an enveloped virus like SARS‐CoV‐2, in liquid with an exposure time of 5 s decreased the titre of AIV by more than 5.2 logs TCID_50_ per ml. Ogilvie et al. (2021) demonstrated that various commercially available alcohol free disinfectants such as benzalkonium chloride (0.2%), Qimei Hand Sanitizing Wipes (0.13% benzalkonium chloride) and Cavicide (0.2% diisobutylphenoxyethoxyethyl dimethyl benzyl ammonium chloride and 17.2% isopropanol) could reduce the SARS‐CoV‐2 titre with an exposure time of 15 s by more than 3.19, 2.97 and 3.19 logs PFU per ml in the absence of soil load, respectively. Although in the presence of 0.5% mucin, these chemicals could reduce the virus titre with an exposure time of 15 s by more than 2.97, 2.64 and 2.97 logs PFU per ml, respectively. Enveloped viruses such as AIV and SARS‐CoV‐2 could be easily inactivated by low concentrations of HClO and benzalkonium chloride. Nevertheless, this is the first report showing drastic reduction of CoVs such as SARS‐CoV‐2 and TGEV in HClO solution by 5 and 6 logs TCID_50_, respectively, and usefulness of HClO to effectively inactivate SARS‐CoV‐2 with low concentration (59.4 ppm), short incubation time (10 s) and soil load (0.1% FBS as a final concentration).

In conclusion, purified HClO, a main component of iPOSH, is a powerful antiviral agent against CoVs including SARS‐CoV‐2. Further studies are necessary to understand real situation of SARS‐CoV‐2 contamination in the environment or clinical setting and whether iPOSH is useful for inactivating SARS‐CoV‐2 in saliva and on different surfaces of various materials.

## CONFLICT OF INTEREST

This study was performed as a collaborative research of Local Power Co., Ltd., and financially supported by this company.
